# Effect of Silica Nanoparticles Blocked with Epoxy Groups on the Crosslinking and Surface Properties of PEG Hydrogel Films

**DOI:** 10.3390/polym13193296

**Published:** 2021-09-27

**Authors:** Junyoung Park, Nahee Kim, Kevin Injoe Jung, Soomin Yoon, Seung Man Noh, Joona Bang, Hyun Wook Jung

**Affiliations:** 1 Department of Chemical and Biological Engineering, Korea University, Seoul 02841, Korea; jypark@grtrkr.korea.ac.kr (J.P.); nahee@grtrkr.korea.ac.kr (N.K.); ij@grtrkr.korea.ac.kr (K.I.J.); 0425soomin@gmail.com (S.Y.); 2 Research Center for Green Fine Chemicals, Korea Research Institute of Chemical Technology, Ulsan 44412, Korea; smnoh@krict.re.kr

**Keywords:** silica nanoparticles, epoxy group, PEG hydrogel, UV crosslinking, rheological properties, surface properties

## Abstract

Silica nanoparticles (G-SiNPs) blocked with 3-glycidoxypropyl trimethoxysilane (GPTS) were newly applied to hydrogel films for improving film coating properties and to distribute the epoxy groups on the film surface. The effects of the content of epoxy-functionalized G-SiNPs on the crosslinking features by photo-induced radical polymerization and the surface mechanical properties of the hydrogel films containing poly(ethylene glycol) dimethacrylate (PEGDMA) and glycidyl methacrylate (GMA) were investigated. The real-time elastic modulus of various PEG hydrogel mixtures with prepared particles was monitored using a rotational rheometer. The distribution of epoxy groups on the crosslinked film surface was directly and indirectly estimated by the elemental analysis of Si and Br. The surface mechanical properties of various hydrogel films were measured by nano-indentation and nano-scratch tests. The relationship between the rheological and surface properties of PEG-based hydrogel films suggests that the use of small amounts of G-SiNPs enhances the surface hardness and crosslinked network of the film and uniformly distributes sufficient epoxy groups on the film surface for further coating applications.

## 1. Introduction

Hydrogels contain three-dimensionally crosslinked polymeric networks capable of absorbing large amounts of water or soluble solvents [1]. Owing to their excellent characteristics (softness, bioactivity, and biocompatibility), they have been widely applied in various industrial fields, such as tissue engineering, drug delivery, sensors, optic devices, and coatings [2].

Among the various hydrogel polymers, poly(ethylene glycol) (PEG) hydrogels have been utilized in many applications because of their attractive features (non-toxicity, high water solubility, and biocompatibility) [3,4]. Representatively, the molecular weight and monomer concentration of the polyethylene glycol dimethacrylate (PEGDMA)-thiol hydrogel system were elaborately tuned by Gavin et al. [5]. The combination of PEGs and biological molecules generally contributes to the elevated biological activity, which increases the resistance of PEG surfaces to cell and protein adsorption [6]. In addition, the structural and gel properties of various PEG hydrogels have been shown to correlate with the gelation dynamics and rheological behavior during ultraviolet (UV) irradiation [7,8,9].

Recently, various studies to impart target-oriented characteristics to PEG hydrogel systems have been attempted by combining functional agents [10,11]. Hybrid PEG hydrogels functionalized with epoxy units have been specifically developed to enhance surface properties for the effective screening of contaminations or corrosions caused by marine organisms on the coating surfaces [12,13]. For example, the ring opening of the epoxy groups on film surfaces can be easily treated using various biomaterials, such as amino acids (cysteine, lysine, and serine) [14]. Certain epoxy groups can be converted to ligands to specifically adsorb the target protein, and the remaining epoxy groups can be transformed into diol groups to reduce nonselective adsorption. Furthermore, an epoxy unit on the film surface can be changed into hydrophobic or hydrophilic brushes in amphiphilic coating fields [15]. Kim et al. [16] investigated the effect of glycidyl methacrylate (GMA) with an epoxy group on the chemo-rheological behaviors of PEG-based hydrogel mixtures. It was observed that a proper portion of GMA contributed to the formation of densely crosslinked networks in PEG hydrogel films, exhibiting enhanced rheological and mechanical properties. However, it was difficult to expose epoxy groups on the film surface in proportion to the amount of the GMA.

A possible strategy for properly distributing epoxy groups onto a hydrogel film surface is to incorporate epoxy-functionalized inorganic nanoparticles in the coating formulation as a building block [17]. In particular, silica nanoparticles (Si-NPs) play an important role as reinforcing extenders in polymeric composites [18,19]. Moreover, attaching any active functional group (amine, carboxyl, thiol, or epoxy) to the Si-NP surface allows the interaction between the inorganic NPs and polymers to tune the surface properties of polymeric composite films [20,21]. Representatively, epoxy-functionalized Si-NPs combined with 3-glycidoxypropyltrimethoxysilane (GPTS) have been elaborately synthesized and demonstrated to exhibit outstanding surface mechanical properties in industrial and commodity products [22,23].

Based on Kim et al. [16], this study attempted to improve the epoxy functionality on the surface of PEG hydrogel films using a small amount of Si-NPs blocked with GPTS as a coupling agent (G-SiNPs). Modified Si-NPs play a key role in exposing the epoxy groups on the film surface to provide the required surface mechanical properties and amphiphilic surface functionality of the hydrogel films. G-SiNPs were directly synthesized through procedures established by Rosen et al. [22] and Chu et al. [23]. A small amount of G-SiNPs was added to PEG hydrogel mixtures containing PEGDMA as a crosslinker, GMA as a monomer, and 2,2-dimethoxy-2-phenylacetophenone (DMPA) as a photo-initiator. The real-time viscoelastic properties of the hydrogel mixtures with an increasing amount of G-SiNPs up to 7 wt% were measured using a rotational rheometer during UV irradiation. The distribution of G-SiNPs on the surface of cured PEG hydrogel films was analyzed by a field emission scanning electron microscope-energy dispersive X-ray spectrometer (FESEM-EDS). The surface roughness caused by the ring opening reaction between the epoxy group and 11-bromoundecanamide with bromine (0.01 M) in an acetone solvent was observed through atomic force microscopy (AFM), confirming that the epoxy group in GMA and G-SiNPs was sufficiently exposed to the surface. The surface mechanical properties of the cured hydrogel films were characterized using nano-indentation and nano-scratch testers.

## 2. Experimental Process

### 2.1. Preparation of Si-NPs Blocked with GPTS

**Figure 1:**
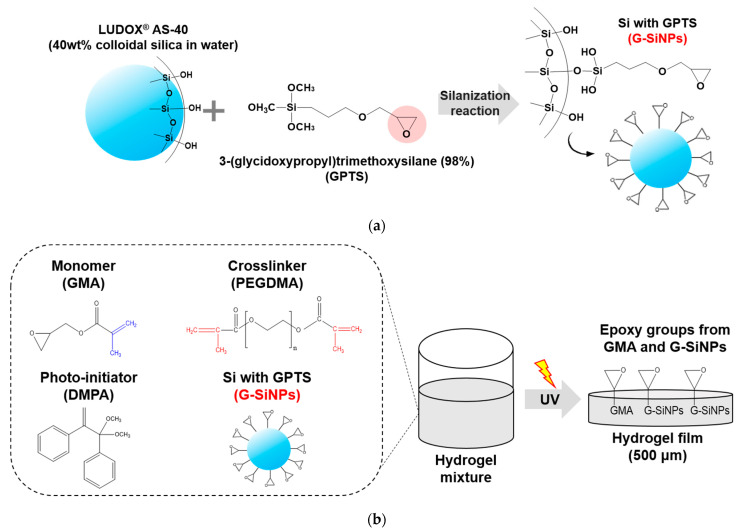
(**a**) Preparation of G-SiNPs by silanization between Si-NPs and GPTS. (**b**) Schematic illustration of a cured hydrogel film with epoxy rings on the surface after UV curing of the hydrogel mixture.

Commercially available LUDOX AS-40 (Si-NPs) with silanol groups were modified using the GPTS containing an epoxy group, following the synthetic procedure introduced by Rosen et al. [22] and Chu et al. [23]. The reaction scheme for producing modified Si-NPs with epoxy groups on the particle surface (G-SiNPs) is illustrated in Figure 1a. For the surface modification of Si-NPs, the Si-OH groups in Si-NPs reacted with GPTS by silanization in an acidic (pH~4) water/ethanol mixture for 24 h at room temperature. The crude G-SiNPs were then purified by the centrifugation, followed by dialysis to completely remove any unreacted GPTS. The final G-SiNP samples were dried under vacuum.

To verify the formation of the G-SiNPs, the chemical changes at the surfaces of the Si-NPs and G-SiNPs were monitored via the attenuated total reflection-Fourier transform infrared spectroscopy (ATR-FT-IR, Agilent Technology, Santa Clara, CA, USA) in transmittance mode. Powder samples were spread on an ATR crystal to record specific IR spectra. In addition, the hydrodynamic diameters of the particles for checking a physical change were measured by dynamic light scattering (DLS, Brookhaven, Holtsville, NY, USA). The 20 mL aqueous solution containing the prepared particles (Si-NPs and G-SiNPs) was illuminated with a laser light source of 365 nm wavelength. The photon collector detected the intensity of the scattered light at an angle of 45°.

### 2.2. Formulation of UV Curable Hydrogel Mixtures

Poly(ethylene glycol) dimethacrylate (PEGDMA, Mw = 550 g/mol) and glycidyl methacrylate (GMA, Mw = 142.15 g/mol) were used as a crosslinker and a monomer, respectively. Based on previous studies [16], the molar ratio for PEGDMA and GMA was fixed at 70:30 mol% (PEG-GMA30) in this study, to exclusively emphasize the role of epoxy groups in G-SiNPs. For the radical polymerization process performed via UV irradiation, 2,2-dimethoxy-2-phenylacetophenone (DMPA) was slightly inserted into the hydrogel mixtures (approximately 1 wt% based on the total weights of PEGDMA and GMA) as a photo-initiator (PI). All materials were purchased from Sigma-Aldrich (St. Louis, MO, USA). Various amounts of G-SiNPs from 0 to 7 wt% (based on the total weights of PEGDMA and GMA) were well dispersed in the PEG-GMA30 hydrogel mixture. The formulations for the hydrogel mixtures are listed in Table 1. Noteworthily, G-SiNP contents exceeding 10 wt% induce severe particle aggregation that degrades the film performance. Figure 1b shows the schematic illustration of a cured hydrogel film with epoxy groups from both GMA and G-SiNPs on the surface through the UV curing process of the hydrogel mixture.

### 2.3. Chemo-Rheological Properties of Hydrogel Mixtures during UV Irradiation

The real-time chemo-rheological behaviors of various UV curable hydrogel mixtures in Table 1 were monitored in terms of the transient storage modulus (G′) and loss modulus (G″) using a rotational rheometer (MCR 302, Anton Paar, Graz, Austria). The measuring gap between the disposable 8 mm upper plate and the lower quartz plate was set at 500 μm. A strain of 1% within the linear viscoelastic region and an input frequency of 5 Hz were applied in the small amplitude oscillatory shear (SAOS) mode. The hydrogel mixtures were irradiated through the lower plate using UV light with a 365 nm wavelength and 1.25 mw/cm^2^ intensity for 5 min [24,25,26].

### 2.4. Surface Analysis of Crosslinked Hydrogel Films

To qualitatively understand the distribution of epoxy groups exposed on the surfaces of crosslinked films of 500 μm thickness produced via rheological tests in Section 2.3, field emission scanning electron microscopy (Quanta 250 FEG, FEI, Hillsbora, OR, USA) with energy-dispersive X-ray spectrometry (FESEM-EDS) analysis was employed under high vacuum conditions and a beam voltage of 10 kV. Using the EDS spectra, the elemental chemical compositions (Si and Br distributions) on the film surface were interpreted [27,28] and were closely related to the distribution of epoxy groups on the surface. Through the FESEM-EDS mapping of Si and Br on the film surface, the amounts of elements relative to the area were quantified. The Si distribution on the cured film surfaces was directly detected. To confirm the Br distribution, a Br solution was prepared using an acetone solvent (0.01 mM of 11-bromoundecanamide with an amine group attached to the end). The crosslinked films were immersed in the Br solution for 24 h for the sufficient ring opening reaction between the amine group in 11-bromoundecanamide and the epoxy group on the surface [29,30]. Subsequently, the films were dried in the vacuum oven for 3 h at 50 °C. Afterward, to indirectly predict the epoxy groups on the film surface, the reaction-induced surface roughness and Br distribution were visualized by atomic force microscopy (AFM wide scan, Anton Paar Tritec SA, Corcelles, Switzerland) and FESEM-EDS, respectively [31,32]. The AFM images were acquired with 256 scan lines for 256 s in a square scan area of 77.1 μm × 77.1 μm. The contact-mode AFM probe scanned the film surface to capture a three-dimensional surface image.

### 2.5. Surface Mechanical Properties of Crosslinked Hydrogel Films

The surface mechanical properties of the cured hydrogel films, such as the indentation hardness and scratch resistance, are strongly dependent on the physical state of the crosslinked films after the curing process [33]. The nano-indentation test (NHT^3^) (Anton Paar Tritec SA, Corcelles, Switzerland) provided the normal force-indentation depth curves of the cured hydrogel films produced by rheological tests in Section 2.3. The normal force was applied at a rate of 2000 nm/min until the Berkovich diamond indenter reached a penetration depth of approximately 3 μm. Thereafter, after a pause of 30 s, the tip was unloaded at the rate of 2000 nm/min from the indented surface. The indentation hardness (HIT), which was determined from the maximum force divided by the calibrated contact area, was evaluated from a force-depth curve, according to the Oliver and Pharr method [34].

The nano-scratch test (NST) (Anton Paar Tritec SA, Corcelles, Switzerland) was employed to evaluate the scratch resistance from the scratch depth profiles [35,36]. A diamond sphero-conical tip horizontally scratched the coating surface along a 1 mm length at the rate of 2 mm/min. During the scratch measurement, the normal force gradually increased from 0.1 to 2 mN. The surface resistance of the cured films was estimated using the penetration depth profiles as a function of the normal force, following standard ASTM D 7187 [37].

## 3. Results and Discussion

### 3.1. Structural Features of G-SiNPs

**Figure 2:**
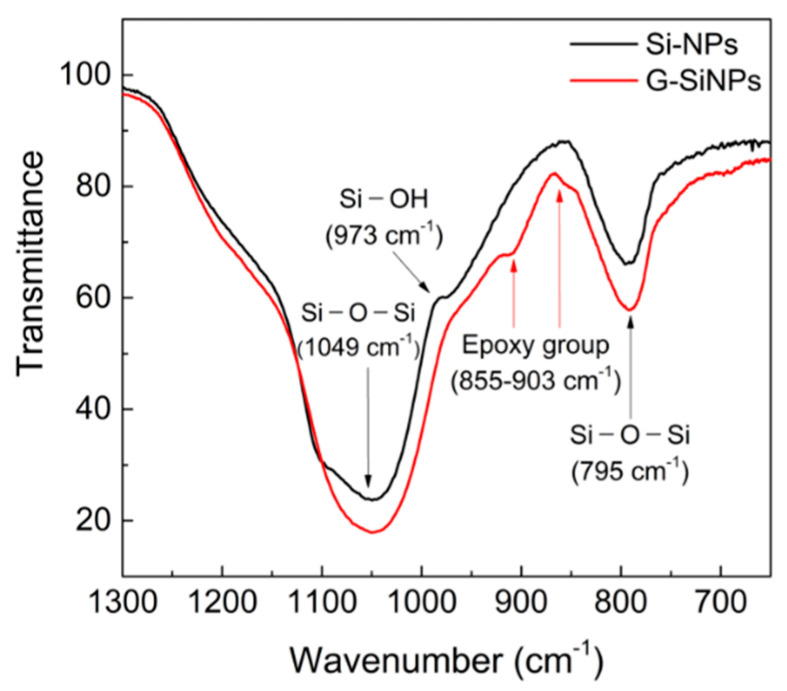
Fourier transform infrared (FT-IR) spectra of SiNPs (black) and G-SiNPs (red).

The structural features of the prepared Si-NPs blocked with GPTS (G-SiNPs) were compared with those of the reference Si-NPs (LUDOX) in the literature [22] using the FT-IR and DLS measurements. Figure 2 shows the FT-IR spectra of the Si-NPs and G-SiNPs along the wavenumber in the range of 650–1300 cm^−1^. The Si-O-Si stretching peak of the colloidal silica particles was observed in both samples at 1049 and 795 cm^−1^. The silanol Si-OH stretching peak in the Si-NPs at 973 cm^−1^ disappeared in that for the G-SiNPs because of the silanization reaction between GPTS and silanol groups on the Si-NP surface. For the G-SiNPs, the peak of the epoxy groups in GPTS attached to the particle surface was newly found within the range of 855–903 cm^−1^ [22,23].

The particle sizes of the Si-NPs and G-SiNPs were quantitatively compared using DLS. Although the diameters measured (25.266 and 35.367 nm for the Si-NPs and G-SiNPs, respectively) were slightly larger than those in the previous study considered (22.9 and 32.2 nm, respectively) [22], the size of the G-SiNPs was observed to be larger than that of the Si-NPs, owing to the silanization reaction [22,38]. It was confirmed from the structural results that the GPTS with the epoxy group was successfully attached to the surface of the Si-NPs.

### 3.2. Real-Time Rheological Properties of Hydrogel Mixtures during UV Irradiation

**Figure 3:**
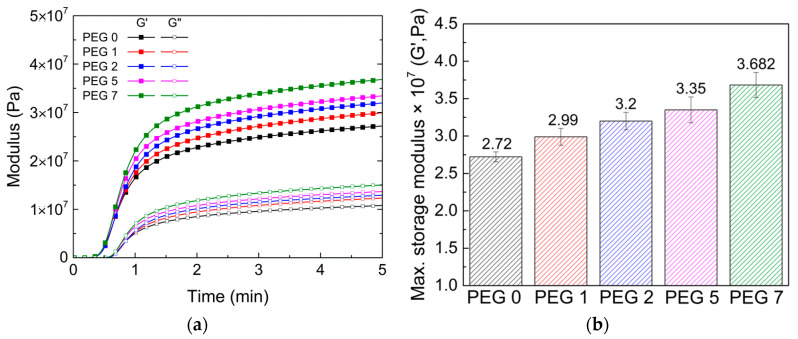
(**a**) Real-time storage modulus (G′) and loss modulus (G″) during ultraviolet (UV) irradiation and (**b**) maximum G′ for the PEG-GMA hydrogel mixtures with varying G-SiNP contents.

To scrutinize the role of epoxy-functionalized G-SiNPs in the formation of crosslinked networks in PEG hydrogel mixtures, the real-time storage modulus (G′) and loss modulus (G″) were monitored during the specified UV curing operation in the SAOS mode. Figure 3a shows the real-time G′ and G″ of the hydrogel mixtures with varying G-SiNP contents. Note that G′ is more focused here because G′ is at a much higher level than G″. In the given sets, the crosslinking between GMA and PEGDMA was practically completed after 1 min of UV irradiation under a UV dose of 1.25 W/cm^2^. As the G-SiNP contents gradually increased, the G′ was slightly enhanced while maintaining the initiation point of the radical polymerization (Figure 3a). The increasing trend of the final G′ values for the hydrogels with increasing G-SiNP content up to 7 wt% is shown in Figure 3b, indicating that an increase in the particle-matrix interfacial area by the epoxy-functionalized nanoparticles improved the viscoelastic properties of the hydrogels [39]. Notably, the G-SiNP content was limited to less than 10 wt% because of unexpected particle aggregation, which can degrade the properties of crosslinked hydrogel films [17].

### 3.3. Distribution of Epoxy Units on Crosslinked Film Surface

**Figure 4:**
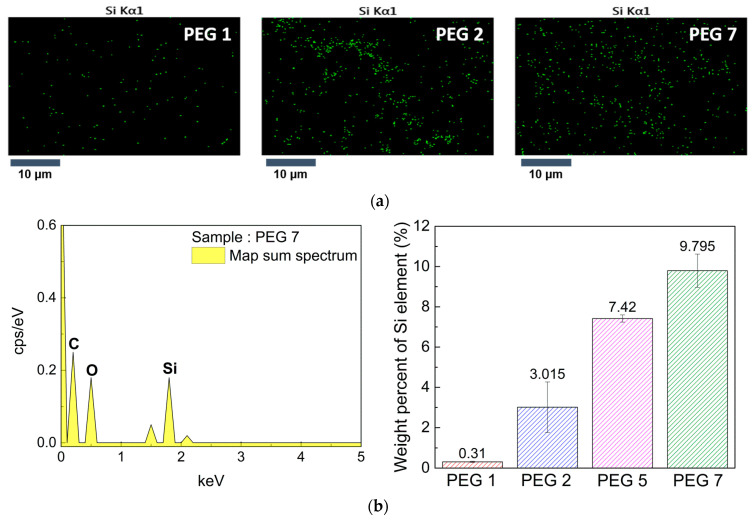
Distribution of Si on the cured film surface by Si mapping for the (**a**) PEG 1, PEG 2, and PEG 7 hydrogel mixtures. (**b**) Integrated area spectra and Si wt% on various crosslinked films with varying G-SiNPs contents obtained by SEM–EDS.

First, FESEM-EDS analysis was performed to qualitatively determine the distribution of epoxy groups in the GMA and G-SiNPs on the post-cured film surface. The mapping images of Si designated as green dots are shown in Figure 4a, confirming that the G-SiNPs in the hydrogels up to 7 wt% content were well dispersed on the film surfaces without aggregation. The integrated area spectra and Si wt% among C, O, and Si on the film surface are shown in Figure 4b. The Si wt% on the film surface proportionally increased with the G-SiNP content in the hydrogel mixtures. It is expected that the distribution of epoxy groups on the film surface can be effectively tuned by adjusting the G-SiNP content, without further increasing the GMA content.

**Figure 5:**
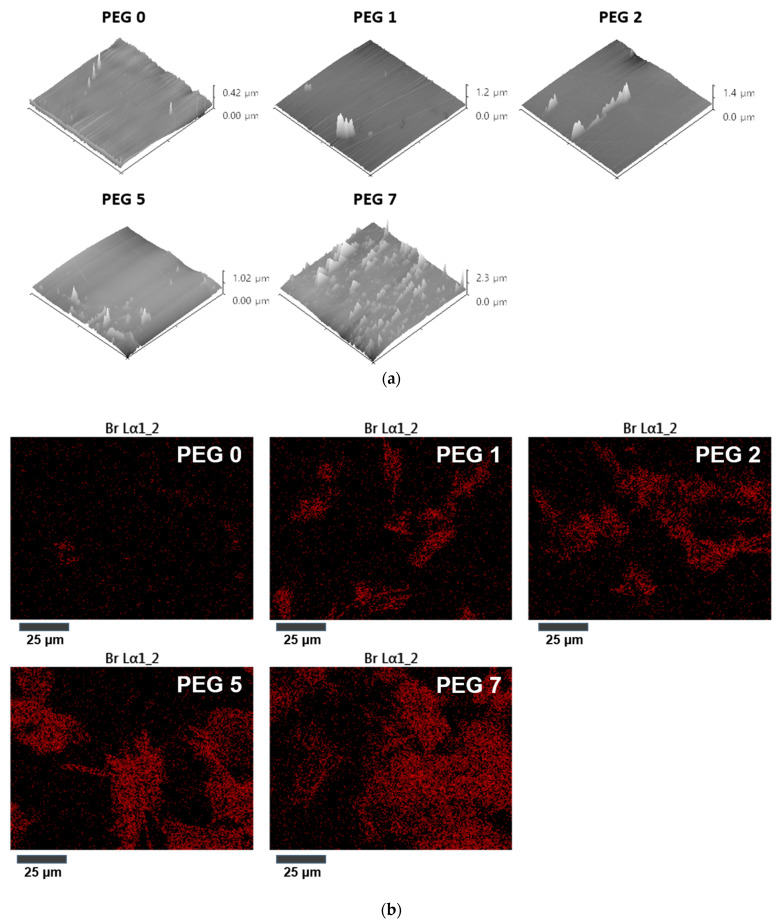

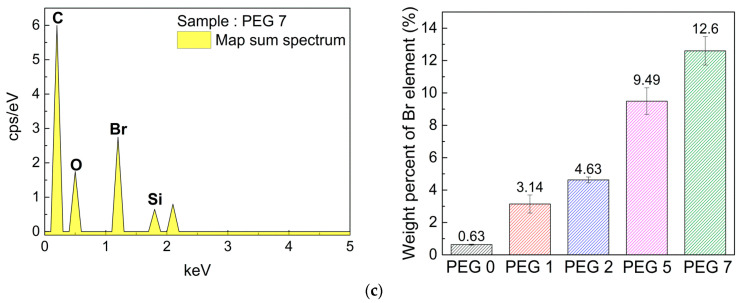
(**a**) Change in the surface roughness after the amine-epoxy ring opening reaction and (**b**) the distribution of Br (Br mapping) on the surface of hydrogel films with varying G-SiNP contents. (**c**) Integrated area spectra and wt% of Br on various crosslinked films with varying G-SiNP contents obtained by FESEM–EDS.

Further experiments were conducted to predict the exposure of epoxy groups (in G-SiNPs or GMA) on the crosslinked film surface by the elemental analysis of Br and the surface roughness after the sufficient amine-epoxy ring opening reaction on the film surface. FESEM-EDS and AFM were employed. As the G-SiNP content increased, the 3D topographical AFM maps treated by the Image J program showed that the surface roughness of the films became more severe (Figure 5a), representing the active ring opening reaction of the epoxy and amine groups with increasing G-SiNP contents up to 7 wt%.

Figure 5b shows the images of the Br elemental distribution on the film surface scanned by FESEM-EDS after the ring opening reaction. The integrated area spectra and Br wt% among C, O, Si, and Br are shown in Figure 5c. Br was evenly distributed on the film surface and the red spots, as the quantitative surface fraction of Br, increased with an increase in the G-SiNP content. These results show that the G-SiNPs excellently distribute epoxy groups on the cured film surface and enhance the crosslinking properties of hydrogel mixtures.

### 3.4. Surface Mechanical Properties of Crosslinked Films

**Figure 6:**
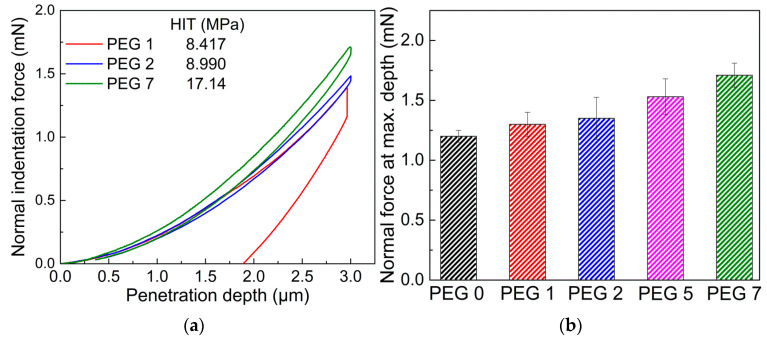
(**a**) Normal indentation force vs. penetration depth profiles and (**b**) maximum normal indentation forces at a 3 μm depth for the cured hydrogel films with varying G-SiNP contents via the nano-indentation test (NHT^3^). The indentation hardness (HIT) values are shown in (**a**).

The surface mechanical properties such as the normal indentation force and scratch resistance were measured on the surfaces of the crosslinked films through nano-indentation (NHT^3^) and nano-scratch (NST) tests [34,35,36]. Normal indentation forces required for an indentation depth of up to 3 μm were compared by NHT^3^, as shown in Figure 6a. As expected from the rheological results, normal indentation forces were gradually enhanced with an increasing G-SiNP content under the given indentation depth condition, owing to the improved elastic modulus and rigidity of the film by the addition of the G-SiNPs. This tendency was observed for the HIT values and maximum normal force data in Figure 6b.

**Figure 7:**
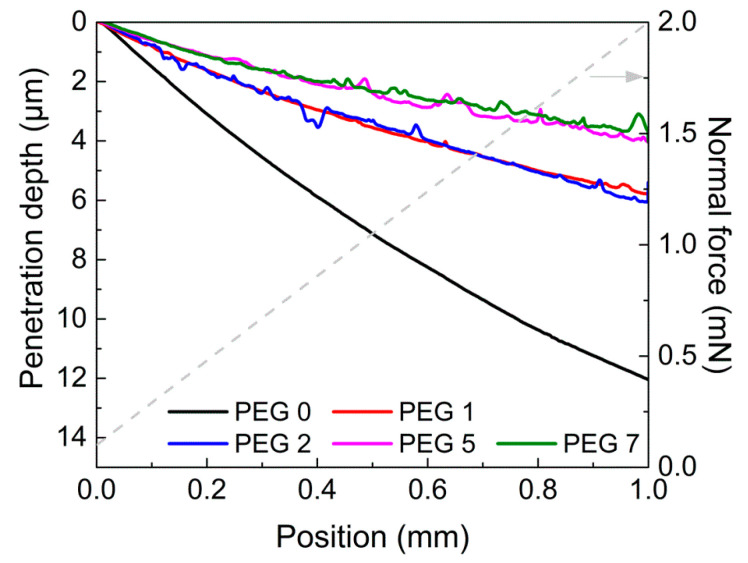
Penetration depth profiles for the cured hydrogel films with different portions of G-SiNPs.

An NST was performed to determine the scratch resistance of the films in terms of the penetration scratch depth by applying a progressive normal force from 0.1 to 2 mN within a 1-mm scanning length. Note that the level of normal force applied to the NST was very similar to the normal indentation force in Figure 6a. Figure 7 shows that the penetration scratch depth became shallower as the G-SiNP content increased. In general, the deeper the penetration, the weaker the scratch resistance of the cured film. As indicated from the nano-indentation data, the scratch resistance was remarkably enhanced by increasing the G-SiNP content, although the penetration depths of PEG 1 (1 wt% of G-SiNPs) and PEG 2 (2 wt%) appeared very similar to each other. The uniform distribution of G-SiNPs as hard materials on the crosslinked film surface could improve the film resistance regarding external loads. Consequently, the addition of a small amount of the G-SiNPs can effectively improve the mechanical properties of the crosslinked films and the distribution of epoxy groups that induce amphiphilic surface properties on the film for further coating applications. Epoxy units on the film surface can effectively impart hydrophilic or hydrophobic coating properties by the selective combination with amphiphilic biomaterials such as amino acids (cysteine, lysine, serine, and so on). For instance, they can play an important role in creating a low-fouling zwitterionic coating surface by reacting with the cysteines for nanomedicine applications [40] and also help to have good stability in bovine serum albumin (BSA) or lysozyme protein solutions [22].

## 4. Conclusions

To improve the surface hardness and sufficiently distribute epoxy groups on hydrogel film surfaces, epoxy-functionalized silica nanoparticles (G-SiNPs) blocked with GPTS were applied to PEG-based hydrogel mixtures. Crosslinking characteristics and surface properties of PEG-GMA30 hydrogel films with different contents of G-SiNPs were experimentally compared. The rheological measurement during the UV curing showed that the real-time rheological properties (i.e., elastic modulus) of PEG hydrogel film were enhanced while maintaining the initiation point of the radical polymerization, as the amount of G-SiNPs was increased up to 7 wt%. The elemental analysis of Si and Br by FESEM-EDS and AFM proved an even distribution of epoxy groups in GMA and G-SiNPs on the hydrogel film surface using G-SiNPs. The effect of the G-SiNPs contents on the surface mechanical properties of various PEG hydrogel films was well interpreted by nano-indentation and nano-scratch tests, representing that the addition of a small amount of G-SiNPs significantly improved the surface mechanical properties such as indentation hardness and scratch resistance. Based on the relationship between the real-time crosslinking characteristics and surface properties of hydrogel films with varying contents of G-SiNPs, the optimal coating formulation and UV curing condition for target-oriented hydrogel films can be favorably tuned. In particular, the epoxy groups exposed on the film surface can be readily functionalized using various biomaterials with amphiphilic properties in novel coating applications.

## Figures and Tables

**Table 1:** Formulation of ultraviolet (UV)-curable PEG-GMA hydrogel mixtures containing synthesized G-SiNPs.

Sample	PEGDMA (g)	GMA (g)	DMPA (g)	G-SiNPs (g)
PEG 0	0.90	0.10	0.01	0.00
PEG 1				0.01
PEG 2				0.02
PEG 5				0.05
PEG 7				0.07

## Data Availability

The data presented in this study are available on request from the corresponding author.
